# Behavioral–Gastrointestinal Interaction Between Night Eating Syndrome and GERD Among Saudi Adults: Implications for Clinical Screening and Lifestyle-Based Care

**DOI:** 10.3390/healthcare14050636

**Published:** 2026-03-03

**Authors:** Mohammad A. Jareebi, Yara A. Mutaen, Hanin J. Mobarki, Alaa A. Faqihi, Shahad A. Ageeli, Rana M. Qaseeri, Walaa A. Ajimi, Norah A. Alhazmi, Saja A. Almraysi, Majed A. Ryani, Farjah H. Algahtani, Ahmed A. Bahri, Abdulwahab A. Aqeeli, Nabeel Mohammed Alkhairat, Adhari A. Alselmi

**Affiliations:** 1 Department of Family and Community Medicine, Faculty of Medicine, Jazan University, Jazan 45142, Saudi Arabia; majedryani@gmail.com (M.A.R.); dr.bahri2010@gmail.com (A.A.B.); aaqeeli@jazanu.edu.sa (A.A.A.); 2 Faculty of Medicine, Jazan University, Jazan 45142, Saudi Arabia; yaramutaen@gmail.com (Y.A.M.); haninmobarki4433@gmail.com (H.J.M.); alaaafaqihi@gmail.com (A.A.F.); shhdqyly90@gmail.com (S.A.A.); m1439q1440@gmail.com (R.M.Q.); wla.ali2003@gmail.com (W.A.A.); nouralhazmii@gmail.com (N.A.A.); sajaalasiri1@gmail.com (S.A.A.); 3 Oncology Center, Chair of Epidemiology and Public Health Research, Faculty of Medicine, King Saud University/King Saud Medical City, Riyadh 12373, Saudi Arabia; falgahtani@ksu.edu.sa; 4 Department of Laboratories and Blood Bank, Jazan University Hospital, Jazan University, P.O. Box 114, Jazan 82817, Saudi Arabia; nmmm-2010@jazanu.edu.sa; 5 Clinical Sciences Department, MBBS Program, Fakeeh College for Medical Sciences, Jeddah 21461, Saudi Arabia; adhari.alselmi@hotmail.com

**Keywords:** gastroesophageal reflux disease, night eating syndrome, eating behavior, dietary habits, lifestyle factors, Saudi Arabia

## Abstract

**Background and Objectives**: Gastroesophageal reflux disease (GERD) is a prevalent gastrointestinal disorder that significantly affects quality of life. Night eating syndrome (NES), characterized by evening hyperphagia and nocturnal eating, may worsen reflux through delayed gastric emptying and increased nocturnal acid exposure, yet evidence in young adults remains limited. This study aimed to estimate the prevalence of GERD and NES, examine their association, and identify clinically relevant predictors among adults in southern Saudi Arabia. **Methods**: A cross-sectional study was conducted among 831 adults (≥18 years) in the Jazan region between November 2024 and April 2025. Data were collected using a validated 54-item online questionnaire assessing sociodemographic characteristics, lifestyle behaviors, dietary habits, and medical history. GERD was defined using the GerdQ (score ≥ 8), and NES using the night eating questionnaire (NEQ ≥ 25). Multivariable logistic and linear regression analyses were performed to identify independent predictors. **Results**: The prevalence of GERD was 25.6%, and 9.7% of participants met the criteria for NES. Higher NEQ scores were independently associated with increased odds of GERD (OR = 1.05, 95% CI: 1.02–1.07; *p* = 0.001). GERD was predicted by tea consumption, middle income (10,000–14,999 SAR), asthma, hiatal hernia, and a family history of GERD. NES was independently associated with GERD, smoking, and frequent intake of fatty foods, chocolate, salty foods, and soft drinks, while male sex, employment or student status, higher income, and fiber-rich food intake were protective. **Conclusions**: NES is significantly associated with GERD among young adults. Integrating screening for disordered eating behaviors and dietary counseling into routine GERD care may improve clinical outcomes.

## 1. Introduction

Gastroesophageal reflux disease (GERD) is a prevalent chronic gastrointestinal disorder characterized by the reflux of stomach acid into the esophagus, resulting from lower esophageal sphincter dysfunction or hiatal hernia [1]. The condition manifests with heartburn, regurgitation, chronic cough, sore throat, and mucosal damage, significantly impairing patients’ quality of life [2]. Global GERD prevalence exhibits considerable geographic variation, ranging from 18% to 28% in North America, 12% in Australia, 2% to 7% in East Asia, up to 20% in Western Asia, 9% to 26% in Europe, and 9% to 33% in the Middle East [2].

In Saudi Arabia, GERD prevalence ranges from 15.2% to 45.5%, with notable regional variation [3,4,5]. Targeted population studies have revealed particularly high rates among specific demographics. For instance, primary healthcare attendees in Abha City demonstrated a prevalence of 67.8%, with half experiencing substantial daily life impairment [6]. Among university students, prevalence rates of 23.1% in Jazan [3] and 23.8% at Shaqra University [1] have been documented. Established risk factors for GERD include analgesic use (particularly nonsteroidal anti-inflammatory drugs), dietary habits, smoking, family history, elevated body mass index (BMI), physical inactivity, and consumption of specific foods, including salt, pickles, and fast food [1,3,4,7].

Night eating syndrome (NES) is an eating disorder characterized by disrupted circadian rhythm of food intake, defined by evening hyperphagia (consuming ≥25% of daily caloric intake after dinner) and/or nocturnal awakenings with food consumption at least twice weekly [8,9]. Additional diagnostic criteria include three or more of the following: morning anorexia, strong urges to eat between dinner and sleep onset, insomnia or prolonged sleep latency, belief that eating facilitates sleep initiation, and depressed mood that worsens in the evening [8]. Global NES prevalence estimates range from 1% to 2% in general populations, with rates of approximately 1.5% in the United States and 1.1% in Germany [10,11]. Among Omani adults, prevalence was 1.5% [11], while a study of medical students in Jeddah, Saudi Arabia, reported 7.3% [12].

The pathophysiological link between NES and GERD is plausible through several mechanisms. Late-night food consumption characteristic of NES delays gastric emptying, prolonging gastric distension and increasing intragastric pressure [13,14]. When individuals adopt a supine position shortly after eating, the anatomical barrier against reflux is compromised, facilitating acid reflux episodes [14,15,16]. Additionally, nocturnal eating disrupts normal circadian patterns of gastric acid secretion and lower esophageal sphincter pressure, potentially exacerbating reflux severity [13,14,17]. Despite this theoretical relationship, empirical evidence remains limited.

To date, only one published study has examined the NES–GERD association. Abed et al. conducted a cross-sectional investigation among Palestinian university students, identifying GERD in 33.4% and NES in 10.3% of participants. Their analysis revealed a significant association between NES and GERD (AOR = 2.84, 95% CI: 1.07–3.19), with night eaters demonstrating substantially elevated GERD risk [18]. However, this single study, conducted exclusively among university students in Palestine, provides insufficient evidence to establish the generalizability of this relationship across different populations and age groups.

Given the scarcity of research examining the NES–GERD relationship and the complete absence of such an investigation in the Jazan region of Saudi Arabia, the present study was undertaken to address this knowledge gap. Specifically, this research aimed to (1) determine the prevalence of GERD and NES among adults in Jazan; (2) examine the association between NES and GERD; (3) identify independent predictors of both conditions; and (4) characterize the sociodemographic, lifestyle, and dietary factors associated with these disorders. Understanding these relationships is essential for developing targeted prevention strategies and informing clinical practice regarding the management of both conditions.

## 2. Materials and Methods

### 2.1. Study Design, Setting, and Period

An analytical cross-sectional study was conducted in the Jazan region, located in southwestern Saudi Arabia, between November 2024 and April 2025. Jazan is a predominantly coastal region with a diverse population including urban and rural residents, providing a representative sample of Saudi adults.

### 2.2. Study Population and Sampling

The target population comprised adults aged 18 years and above residing in the Jazan region. A convenience sampling approach was employed to recruit participants through social media platforms and community networks. Inclusion criteria were (1) age ≥ 18 years; (2) current residence in Jazan region; (3) ability to read and comprehend Arabic; and (4) willingness to provide informed consent. Exclusion criteria included (1) incomplete questionnaire responses; (2) pregnancy (due to physiological GERD common in pregnancy); and (3) diagnosed with psychiatric disorders that might influence eating behaviors independent of NES.

Participants with previously diagnosed psychiatric disorders known to significantly influence eating behavior independent of night eating syndrome were excluded. These included clinically diagnosed major depressive disorder, bipolar disorder, schizophrenia spectrum disorders, generalized anxiety disorder, and formally diagnosed eating disorders such as binge eating disorder, bulimia nervosa, or anorexia nervosa. Psychiatric status was determined based on self-report of prior medical diagnosis. No formal psychiatric screening instrument was administered. Therefore, misclassification cannot be excluded, and undiagnosed or unreported psychiatric conditions may have remained within the sample.

### 2.3. Sample Size Calculation

Sample size was calculated using Raosoft calculator (www.raosoft.com/samplesize.html, accessed on 13 November 2024) [19]. Based on the adult population of Jazan (estimated at 919,267) [20], with a 95% confidence level, 50% expected response distribution (most conservative estimate), and 5% margin of error, the minimum required sample was 384 participants. Accounting for a 25% non-response rate, the target sample size was adjusted to 481 participants. The study successfully recruited 831 participants, exceeding the required sample size and enhancing statistical power.

### 2.4. Data Collection Instrument

Data were collected using a self-administered online questionnaire developed in Arabic and distributed via Google Forms. Using convenience sampling, the survey was implemented online. The survey link was posted on social media, mainly on WhatsApp and Facebook, and was shared in professional networks and personal contacts. The questionnaire comprised 54 items organized into four sections:

Section 1: Sociodemographic characteristics including age, sex, nationality, residence (urban/rural), marital status, occupation, educational level, and monthly family income.

Section 2: Lifestyle and health-related factors including anthropometric measurements (self-reported weight and height for BMI calculation), khat use (Catha edulis, a flowering evergreen shrub chewed for stimulant effects), smoking status, physical activity level, dietary habits (meal frequency, consumption patterns of fatty foods, spicy foods, chocolate, fiber-rich foods, salty foods, pickles, coffee, tea, milk, soft drinks, herbal products), eating speed (less than 20 min), and chronic disease history (diabetes mellitus, hypertension, asthma, sickle cell disease, thalassemia, hiatal hernia, otitis media). For females, pregnancy history and GERD during pregnancy were assessed.

Section 3: GERD assessment was undertaken using the validated GERD questionnaire (GerdQ) [21]. This six-item instrument evaluates heartburn frequency, regurgitation, epigastric pain, nausea, sleep disturbance due to reflux, and use of over-the-counter medications. Responses are scored on a 4-point Likert scale. Total scores range from 0 to 18, with scores ≥ 8 indicating probable GERD with 65% sensitivity and 71% specificity. Additional GERD-related variables included self-reported diagnosis, family history of GERD, heartburn presence, proton pump inhibitor use, and analgesic consumption frequency. Given the moderate sensitivity and specificity of GerdQ, misclassification of both false positives and false negatives is possible. Consequently, prevalence estimates derived from the GerdQ should be interpreted as indicative of symptom-defined reflux rather than definitive physician-confirmed GERD.

Section 4: NES assessment, undertaken using the validated NEQ [22]. This 14-item instrument evaluates evening hyperphagia, nocturnal eating frequency, morning anorexia, sleep onset delay, mood disturbances, and awareness during eating episodes. Items are scored on a 5-point Likert scale. Total scores range from 0 to 52, with scores ≥ 25 indicating probable NES. Additional NES-related variables included self-reported NES awareness, symptom duration, and level of consciousness during eating episodes. NEQ score was entered as a continuous predictor to preserve statistical power and avoid arbitrary categorization. The model therefore assumes a linear association between NEQ score and the log-odds of GERD.

### 2.5. Data Analysis

Data was analyzed using R software (version 4.2.3; R Foundation for Statistical Computing, Vienna, Austria). Continuous variables were presented as means ± standard deviations and categorical variables as frequencies and percentages. Multiple linear regression was performed to identify predictors of NES scores (continuous outcome), while multiple logistic regression examined predictors of GERD status (binary outcome: GerdQ score ≥ 8 vs. <8).

Model building followed a theory-driven approach based on clinical relevance and prior literature. All key sociodemographic factors, lifestyle behaviors, dietary habits, and chronic disease variables were entered simultaneously into multivariable models to control potential confounding, and no automated stepwise selection procedures were used. For presentation clarity, only statistically significant predictors are displayed in the main tables, while the models were estimated including all covariates listed.

This approach was chosen to ensure adjustment for theoretically relevant confounders. Variables were retained based on statistical significance (*p* < 0.05) and evaluation of potential confounding effects.

Multicollinearity was assessed using variance inflation factors (VIFs). All VIF values were below the commonly accepted threshold of 5, indicating no evidence of problematic multicollinearity. However, given the conceptual relatedness of certain socioeconomic variables (income, education, and occupation), some degree of shared variance is possible.

The logistic regression model included 213 GERD events. A total of 41 independent parameters were entered into the final multivariable model. The events-per-variable (EPV) ratio was therefore approximately 5.2. While this is below the traditional recommended threshold of 10 events per parameter, all predictors were included based on theoretical and clinical relevance rather than automated selection procedures.

For linear regression models, assumptions of normality and homoscedasticity were assessed through residual diagnostics. Model fit was evaluated using adjusted R^2^ for linear regression models and pseudo-R^2^ for logistic regression models. Statistical significance was defined as *p* < 0.05.

### 2.6. Ethical Considerations

The study received ethical approval from the Jazan University Standing Committee for Scientific Research (approval number: REC-46/06/1285) on 24 December 2024. The study was conducted in accordance with the Declaration of Helsinki and adhered to the STROBE guidelines for cross-sectional studies. Electronic informed consent was obtained from all participants prior to questionnaire completion, following comprehensive explanation of the study purpose, procedures, confidentiality measures, voluntary participation, and right to withdraw. No personal identifiers were collected, ensuring participant anonymity. Data access was restricted to the research team members only.

## 3. Results

### 3.1. Sociodemographic Profile

This study comprised 831 participants with a mean age of 30.64 ± 12.65 years. The majority of the participants were females, who constituted 55.6% of the sample. More than half of the participants (58%) resided in rural areas, 59% were single, and 52% were students. Regarding educational attainment, the majority of participants (72%) held diplomas or bachelor’s degrees. Monthly family income distribution showed that 38% of participants earned more than 15,000 SAR (Table 1).

### 3.2. Health and Habitual Related Variables

Among the 831 participants, mean BMI was 25 ± 6.2 kg/m^2^. Current khat users comprised 2.9% of the sample, while current smokers accounted for 5.1%. Nearly half of the participants (48.1%) reported no regular physical activity during the week (Table 2).

Dietary habits varied considerably among participants (Table 3). Approximately 46% consumed two meals per day. Regular consumption of fatty foods was reported by 62% of participants, spicy foods by 54%, and chocolate by 56%. Fiber-rich foods were consumed by 59%, and salty foods by 55%. The majority (75%) did not consume pickles, and only 7% followed a vegetarian diet (Table 3).

Among the 462 female participants, 357 (77.3%) had experienced pregnancy, of whom 97 (27.2%) reported GERD during pregnancy. Regarding chronic disease prevalence, the majority of participants (>90%) had not been diagnosed with chronic conditions, including hypertension, asthma, or diabetes (Table 4).

### 3.3. Prevalence of GERD, NES and Related Variables

GERD Prevalence

The mean GERD score among participants, as assessed by the GerdQ questionnaire, was 6.59 ± 1.86 (range: 0–18). Based on the validated cutoff score of ≥8, 213 individuals (25.6%) were classified as having probable GERD. When participants were asked about previous clinical diagnosis of GERD by a healthcare provider, 148 (18%) reported having been diagnosed with the condition. Regarding symptom management, 188 participants (23%) reported current or prior use of proton pump inhibitors (PPIs) (Table 5).

NES Prevalence

Objective assessment using the NEQ revealed that 81 participants (9.7%) met the diagnostic criteria for NES (score ≥ 25). In contrast, only 36 participants (4.3%) reported a self-reported NEQ-defined NES, despite a larger proportion meeting the questionnaire-based criteria.

Among the 81 participants meeting objective NES criteria (NEQ ≥ 25), only 4 (4.9%) had self-reported awareness of their condition. Thirty-two participants self-reported NES despite not meeting objective diagnostic criteria (NEQ score < 25). The mean NES score among all participants was 16.34 ± 7.09 (Table 6).

### 3.4. Prevalence of GERD Among NES Patients

**Figure 1:**
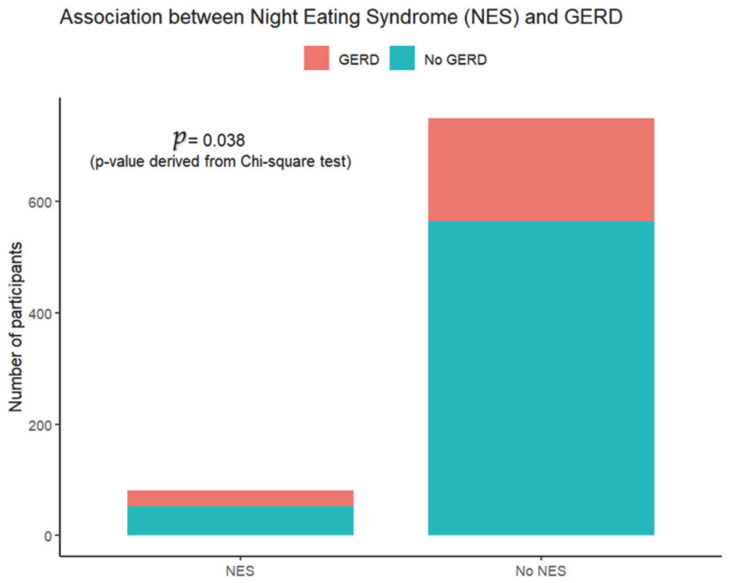
Prevalence of GERD in patients with NES.

The prevalence of GERD was higher among individuals diagnosed with NES compared with those without NES (35.8% vs. 24.5%) (Figure 1). This difference was statistically significant, indicating a positive association between NES and GERD in the study population (*p* = 0.038).

### 3.5. NES Predictors Among Study Sample

Multiple linear regression analysis was conducted to identify factors associated with NES scores. Significant positive association was found with higher NES scores included GERD diagnosis (β = 1.89), current smoking (β = 2.64), diploma or bachelor’s degree education (β = 1.30), and consumption of fatty foods (β = 1.20), chocolate (β = 1.03), salty foods (β = 1.11), and soft drinks (β = 1.23). In contrast, significant protective factors associated with lower NES scores included male sex, employment, student status, higher monthly income exceeding 15,000 SAR, and regular consumption of fiber-rich foods (*p* < 0.001) (Table 7). The overall model explained 19.1% of the variance in NES scores (R^2^ = 0.191, adjusted R^2^ = 0.151).

### 3.6. GERD Predictors Among Study Sample

Multiple logistic regression analysis was performed to identify independent predictors of GERD as defined by a GerdQ score ≥ 8. The NEQ score showed a significant positive association with GERD risk (OR = 1.05, 95% CI: 1.02–1.07, *p* = 0.001). Participants with a family income between 10,000 and 14,999 SAR (OR = 2.02) and those reporting tea consumption (OR = 1.51) had significantly higher odds of GERD. Among medical comorbidities, asthma (OR = 1.79), a family history of GERD (OR = 1.67, 95% CI: 1.13–2.44, *p* = 0.009) and hiatal hernia emerged as the strongest predictors of GERD (OR = 3.61) (Table 8). The logistic regression model demonstrated a modest discriminatory ability with an R^2^ of 0.132.

## 4. Discussion

### 4.1. Principal Findings

This cross-sectional investigation among 831 adults in Jazan, Saudi Arabia, found a GERD prevalence of 25.6% and NES prevalence of 9.7%, with a significant positive association between both conditions. Regression analyses identified distinct yet overlapping predictors, suggesting a complex relationship between GERD and NES that may warrant targeted clinical assessment and management.

### 4.2. GERD Prevalence and Comparison with Previous Studies

The GERD prevalence observed in this study aligns with previously reported Saudi estimates (15.2–45.5%) [3,4,5] and those from broader Middle Eastern countries (9–33%) [2]. It closely matches values reported at Jazan University (23.1%) [3] and Shaqra University (23.8%) [1], as well as estimates from Western countries (10–20%) [23]. In contrast, primary care attendees in Abha presented with higher prevalence (67.8%) [6]. This may be attributed to the high prevalence of unhealthy habits among students and young adults in the region, including high fast-food consumption, irregular meals, and insufficient intake of fruits and vegetables [24,25].

In our study, only 18% of participants reported a physician diagnosis of GERD. Similarly, in clinical populations undergoing upper gastrointestinal endoscopy, approximately 20.1% were diagnosed with GERD [26]. Large population-based surveys have reported that only about 2% of individuals had a prior physician diagnosis, most of whom received treatment [27]. Prevalence estimates vary substantially depending on case definition; symptom-based studies often report rates exceeding 20–30%, whereas physician-diagnosed GERD in the general population is typically lower. GERD is frequently underdiagnosed due to both behavioral and clinical factors. Many individuals perceive heartburn and regurgitation as benign, intermittent symptoms or attribute them to dietary causes rather than a chronic condition, leading to delayed healthcare seeking and underestimation of disease burden. Self-treatment with over-the-counter antacids further contributes to under recognition and misclassification. Clinically, GERD encompasses a broad spectrum of manifestations beyond classic heartburn, including atypical or extra-esophageal symptoms such as chronic cough or hoarseness, which may not be readily attributed to reflux by patients or clinicians, resulting in missed diagnoses [28].

### 4.3. NES Prevalence and Awareness Deficit

The NES prevalence markedly exceeds global estimates of 1–2% [8,9] and is higher than values from the United States, and Germany (all ~1.5%) [10,11]. Our findings more closely resemble those from Jeddah medical students (7.3%) [12] and Palestinian university students (10.3%) [18]. This high prevalence in the Arab population has been associated with rapid sociocultural changes, including high obesity rates (roughly 70% of the population has a BMI > 25), and high levels of stress, anxiety, or depression. Additionally, cultural patterns such as late meals, social eating norms, and irregular sleep schedules may contribute to elevated NES prevalence in Saudi populations [29].

A notable finding is the awareness gap: 95.1% of individuals meeting NEQ criteria were unaware of having NES, while several self-reported NES without meeting diagnostic criteria. A survey of college students in Manipur found that 61.5% of respondents lacked awareness about NES and its consequences [29]. Research on NES more broadly confirms that it is an under-represented and under-studied eating disorder, contributing to limited dissemination of information among both health professionals and the general public [8].

The awareness gap in NES is present for multiple reasons. First, the syndrome has historically been under-recognized in diagnostic manuals and clinical training, which limits its visibility among healthcare providers and the public [8]. Second, there exists mixed consensus and variability in diagnostic criteria and assessment tools across studies, which complicate public and professional understanding [30]. Nevertheless, among individuals who are aware of NES, behavioral change is not automatic; awareness does not invariably translate into cessation of night eating, especially when maladaptive eating habits are maintained by psychological distress, circadian rhythm disruptions, or emotional regulation difficulties [8]. To overcome awareness gaps, a multifaceted approach prioritizing research, clinical consensus, and public health education is essential [22]. Given NES associations with obesity, metabolic syndrome, mood disorders, and sleep disturbance [8,9], clinicians should maintain high suspicion when encountering such comorbidities.

### 4.4. Association Between NES and GERD

The positive NES/NEQ–GERD association supports a significant relationship between nighttime eating and reflux. Although the per point increase in risk appears modest, the cumulative effect across the full range of NEQ scores may be clinically meaningful, such that individuals meeting the NEQ-defined diagnostic threshold for NES could exhibit substantially higher odds of GERD. This aligns with Abed et al., who reported a stronger association (AOR = 2.84) among Palestinian students [18]. Previous studies suggested that late-night eating might delay gastric emptying, increase intragastric pressure, and promote reflux when individuals lie down shortly after eating [13,14,15,16]. Delayed eating may demonstrate delays in metabolic hormones, including leptin and insulin, with ghrelin rhythms shifting, resulting in disrupted homeostatic signaling and circadian dysynchrony. Because circadian clocks regulate gastric motility and lower esophageal sphincter (LES) function, eating at biologically inappropriate times may exacerbate reflux propensity [31]. Along with circadian clock disruption, elevated stress and altered cortisol rhythms can affect LES tone and gastric motility [17,32,33].

Obesity has also been identified as a potential contributor to GERD. Increased intra-abdominal pressure in obesity can impair LES function, increase transient LES relaxations, and predispose individuals to hiatal hernia. Evidence suggests that a BMI reduction of ≥3.5 units may reduce the risk of frequent GERD by nearly 40%. While NES is cross-sectionally associated with GERD, high BMI is not always an independent predictor when NES is considered; however, the coexistence of obesity and night eating may create a higher-risk profile for reflux [18]. In the present study, non-linear associations or threshold effects were not formally assessed. It remains possible that the association is stronger among individuals exceeding the NEQ diagnostic cutoff (≥25), which warrants further investigation using flexible modeling approaches.

### 4.5. Predictors of GERD

In our study, tea consumption, residence in a middle-income group, and clinical factors, including hiatal hernia, asthma, and family history of GERD, have emerged as potential predictors. Previous studies report strong associations for hiatal hernia (72 to 96%) [34], asthma (15 to 82%) [35], and positive family history (41 to 45%) [36,37]. Mechanistically, hiatal hernia promotes reflux by disrupting the anti-reflux barrier at the gastroesophageal junction, reducing LES pressure and impairing diaphragmatic support, thereby increasing esophageal acid exposure [38]. In asthma, recurrent coughing and heightened negative intrathoracic pressure augment the transdiaphragmatic pressure gradient, predisposing to transient lower esophageal sphincter relaxations and facilitating reflux [39].

Regarding tea consumption, prior meta-analytic evidence has not demonstrated a consistent association with GERD. However, experimental and observational data, including findings by Cao et al., suggest that high tea intake may induce transient LES relaxation and increase gastric acidity, potentially mediated by theophylline. Nonetheless, tea consumption may also reflect broader dietary patterns or meal timing behaviors rather than serving as an independent causal factor [40].

Residence in a middle-income group was independently associated with GERD in our analysis, although it is infrequently reported as a direct predictor. This relationship may reflect rapid urbanization, westernized dietary patterns, obesity trends, and shifting socioeconomic conditions, particularly in Middle Eastern and African populations undergoing epidemiological transition. Underdiagnosis related to limited awareness and healthcare access may further obscure the true burden [41]. Temporal trend analyses from these regions indicate a gradual annual increase in GERD prevalence of approximately 0.3% [42].

### 4.6. Predictors of NES

In our analysis, female sex, smoking, higher educational attainment, higher income, employment, and student status were identified as predictors of NES. Dietary factors further demonstrated increased risk with higher consumption of fatty foods, chocolate, salty foods, and soft drinks, whereas fiber intake appeared protective. Although some studies report no significant sex differences [43], a substantial body of evidence indicates greater female vulnerability. Certain investigations have documented nearly double the prevalence among women compared with men, and a predominance of female cases, often accompanied by higher BMI, depressive symptoms, and insomnia [44]. Experimental data also suggest that females may exhibit heightened stress responsivity and emotional dysregulation, which are recognized triggers of nocturnal eating behaviors [45].

Evidence regarding educational level and income remains inconsistent. Some population-based cohorts, including Australian samples, have linked NES to lower educational attainment and lower household income [46]. In contrast, Elsahoryi et al. have reported a greater symptom burden among individuals with higher education or enrollment in non-medical academic programs. University students represent a particularly vulnerable subgroup due to academic stress, irregular sleep cycles, financial strain, and social influences that may destabilize circadian eating patterns [44]. Although a direct causal association between higher income and NES has not been firmly established, greater household income may indirectly shape dietary behaviors through increased consumption of energy dense foods, animal protein, and sugars, as well as more frequent eating outside the home, thereby promoting obesity and dysregulated eating patterns [47]. Smoking, both current and former, has consistently been associated with elevated risk [44]. Nicotine acts as a central stimulant that disrupts sleep architecture, suppresses appetite during daytime hours leading to compensatory nocturnal intake, and is associated with heightened stress and anxiety, all of which may facilitate the development or persistence of night eating behaviors [48].

### 4.7. Clinical Implications

Given the observed association of NES and GERD, clinicians may consider assessing co-occurring symptoms. Clinicians may consider evaluating reflux symptoms in individuals presenting with NES to ensure comprehensive assessment of co-occurring conditions. Assessment of meal timing and dietary patterns may be clinically relevant in patients with GERD, particularly when NES-related behaviors are present [7,49]. However, given the cross-sectional design of the study, the observed association between NES and GERD should not be interpreted as causal. The presence of unmeasured confounding factors, such as obesity, sleep disturbances, or dietary composition, could contribute to the observed association, and should be carefully controlled for future investigations. More than 95% of those afflicted with NES are unknown to this disorder, demonstrating the need for increased advocacy for medical professionals to probe for NES. High-risk individuals, especially the obese, those with metabolic syndrome, and individuals suffering from sleep disorders and mood disorders, should be the focus of public health campaigns.

Consumption of high-fiber foods, structured meal timing and modification of nocturnal eating patterns may be considered as part of broader symptom management strategies, although longitudinal studies are required to determine temporal relationships. In addition, reducing processed foods and foods high in fats, sugars, and calories are just some of the many behaviors that can be modified to implement a variety of interventions. In these individuals, a patient-centered, multidisciplinary approach addressing psychological distress, routine stabilization, and access to healthier food options may be considered as part of comprehensive care. These findings elucidate the need for continued multidisciplinary care that integrates nutrition, behavior, sleep, and lifestyle changes for individuals diagnosed with GERD, NES, or both.

### 4.8. Strengths and Limitations

Key strengths include the large sample size (n = 831), use of validated questionnaires (GerdQ, NEQ), and comprehensive assessment of sociodemographic, lifestyle, dietary, and clinical variables, enabling robust multivariable modelling. The diversity of the sample enhances the representation of adults in Jazan. However, limitations warrant acknowledgment. The cross-sectional design precludes the establishment of temporal or causal relationships between NES and GERD. Due to potential reporting bias, self-reported variables such as height, weight, dietary intake, GERD status, and lifestyle behaviors including smoking and night eating may be subject to differential misclassification. For example, participants experiencing more severe GERD may recall or report their dietary patterns and nocturnal eating behaviors differently compared with those without GERD. In addition, several identified predictors, including tea consumption, income level, and educational attainment, may act as proxies for broader unmeasured constructs, such as overall dietary patterns, psychosocial stress, sleep behavior, or urbanization. Residual confounding cannot be excluded, and these associations should not be interpreted as mechanistic or causal. Although validated instruments were used, objective measures (e.g., endoscopy, polysomnography) were not available.

Additionally, several questionnaire components assessing detailed dietary patterns and lifestyle behaviors were self-constructed and lacked prior psychometric validation, which may have introduced measurement error and reduced the reliability and predictive precision of these variables in regression analyses. Moreover, the use of online convenience sampling may have introduced selection bias. Individuals who are younger, more educated, more digitally literate, or health conscious are more likely to engage with and complete online surveys. Consequently, certain population subgroups, including older adults, individuals with lower educational attainment, limited internet access, or reduced health awareness, may be underrepresented. As a result, the findings may not be fully generalizable to the broader adult population of the Jazan region, particularly older individuals, those with limited digital access, or populations with differing sociodemographic or healthcare utilization profiles. The female-skewed sample (55.6%) and predominantly Saudi composition (98%) may limit generalizability to males and to individuals from other national or cultural backgrounds, where lifestyle, dietary habits, and healthcare access may differ.

Psychiatric exclusions were based on self-reported prior diagnoses rather than structured clinical assessment or medical record verification. This approach may have resulted in residual confounding due to underreporting or undiagnosed psychiatric conditions. Modest model explanatory power (adjusted R^2^ = 0.151 for NES; R^2^ = 0.132 for GERD) suggests that unmeasured psychological, genetic, or environmental factors likely play substantial roles. Due to the moderate sensitivity and specificity of GerdQ, there is a possibility of non-differential misclassification, which may have influenced prevalence estimates and attenuated or inflated observed associations. Seasonal variation could also influence eating and sleep behaviors, as the study was conducted over a defined six-month period. NEQ score was modeled as a continuous linear predictor in logistic regression, and non-linear functional forms.

The study included 213 GERD cases; however, the number of covariates entered into the logistic regression model resulted in an EPV ratio of approximately 5.2. This is below the commonly recommended threshold of 10 and may increase the risk of overfitting, potentially affecting the stability and generalizability of the regression coefficients. Consequently, effect estimates should be interpreted cautiously. VIF were below conventional thresholds, the study included conceptually related socioeconomic indicators (income, education, and occupation), which may share overlapping variance. Future analyses with larger event counts or use of penalized regression techniques (e.g., LASSO) are warranted. Using dimensional reduction or penalized regression approaches may further clarify the independence of these predictors. Despite these limitations, this study provides the first comprehensive analysis of the NES–GERD relationship in the Jazan region and generates evidence to inform clinical decision-making and guide future longitudinal research.

## 5. Conclusions

This study identified a substantial prevalence of GERD (25.6%) and NES (9.7%) among adults in Jazan, with a statistically significant positive association between the two conditions. Each 1-point increase in NEQ score was associated with a 5% increase in the odds of GERD. GERD showed statistically significant associations with tea consumption, asthma, hiatal hernia, and middle-income level. These associations likely reflect complex behavioral and socioeconomic correlations rather than direct mechanistic effects. In contrast, NES was associated with GERD, smoking, and high-calorie food intake, and showed inverse associations with male sex, employment, higher income, and fiber-rich dietary patterns. These patterns may reflect overlapping behavioral, dietary, and sociodemographic correlations rather than direct mechanistic pathways. Notably, approximately 95% of individuals meeting criteria for NES were unaware of their condition, highlighting important gaps in recognition and public awareness. The observed co-occurrence of GERD and NES suggests that clinicians may consider assessing both conditions to support comprehensive care. However, given the cross-sectional design, these findings should be interpreted as associative rather than indicative of temporal or causal relationships. Longitudinal and mechanistic studies are required to determine directionality, clarify underlying pathways, and evaluate whether modification of associated behaviors alters symptom trajectories.

## Figures and Tables

**Table 1:** Sociodemographic characteristics of study participants (n = 831).

Characteristic	N	%
**Age (years),** Mean ± SD	30.64 ± 12.65	
**Sex**		
Male	369	44
Female	462	56
**Nationality**		
Saudi	818	98
Non-Saudi	13	2
**Residence**		
Urban	349	42
Rural	482	58
**Marital Status**		
Single	492	59
Married	322	39
Divorced/widowed	17	2
**Occupation**		
Unemployed	95	12
Student	434	52
Employed	302	36
**Education**		
Secondary school or less	211	25
Diploma or bachelor’s degree	596	72
Postgraduate studies	24	3
Monthly Family Income (SAR) *		
Less than 5000 (<1333 USD)	189	23
5000–9999 (1333–2666 USD)	175	21
10,000–14,999 (2667–4000 USD)	154	18
More than 15,000 (>4000 USD)	313	38

SD: Standard deviation; SAR: Saudi Riyal. * 1 USD ≈ 3.75 SAR.

**Table 2:** Anthropometric and habitual characteristics (n = 831).

Characteristic	N	%
**Weight (kg), mean ± SD**	66 ± 19	
**Height (cm), mean ± SD**	162 ± 9.1	
**BMI (kg/m^2^), mean ± SD**	25 ± 6.2	
**Khat Use**		
No	770	93
Previous user	37	5
Current user	24	3
**Smoking Status**		
No	724	87
Previous smoker	65	8
Current smoker	42	5
**Physical Activity**		
≥30 min, 5 times/week	213	26
<30 min, 5 times/week	218	26
No physical activity	400	48

*SD: Standard deviation; BMI: Body mass index.*

**Table 3:** Dietary habits of study participants (n = 831).

Characteristic	N	%
**Fast Eating (Less than 20 min)**	453	55
**Meal Frequency (per day)**		
One meal	56	56
Two meals	384	46
Three meals	343	41
More than three meals	48	6
**Food Type**		
Fatty food	516	62
Spicy food	449	54
Chocolate	467	56
Fiber-rich foods	489	59
Salty foods	461	55
Pickles	208	25
Vegetarian diet	61	7
Coffee	526	63
Tea	493	59
Milk	353	42
Soft drinks	383	46
Herbs	176	21

**Table 4:** Chronic diseases among participants (n = 831).

Characteristic	N	%
Diabetes mellitus	60	7
Hypertension	65	8
Asthma	74	9
Sickle cell disease	24	3
Thalassemia	14	2
Hiatal hernia	9	1
Otitis media	64	8
GERD during pregnancy (n = 357) *	97	27

* Among 357 females who had been pregnant (77.3% of 462 female participants).

**Table 5:** GERD related variables (n = 831).

Characteristic	N	%
GERD score, mean ± SD	6.59 ± 1.86	
GERD (based on GerdQ score ≥ 8)	213	26
GERD (self-reported diagnosis)	148	18
PPI Use	188	23

*SD: Standard deviation; GerdQ: GERD questionnaire; PPI: Proton pump inhibitor.*

**Table 6:** NES-related variables (n = 831).

Characteristic	N	%
NES score, mean ± SD	16.34 ± 7.09	
NEQ-defined NES (NEQ score ≥ 25)	81	10
NES (self-reported)	36	4
**Level of Awareness**		
Fully unaware	514	62
Somewhat unaware	36	4
Somewhat aware	72	9
Largely aware	59	7
Fully aware	150	18

*SD: Standard deviation; NEQ: Night eating questionnaire.*

**Table 7:** Determinants of NES score: Multiple linear regression *.

Variables	β	95% CI	*p*-Value
**Risk Factors**			
GERD diagnosis	1.89	0.81–2.97	0.001
Diploma/bachelor’s degree	1.30	0.17–2.44	0.024
Current smoking	2.64	0.41–4.88	0.020
Fatty food consumption	1.20	0.17–2.23	0.022
Chocolate consumption	1.03	0.01–2.05	0.047
Salty foods	1.11	0.13–2.08	0.026
Soft drinks	1.23	0.22–2.23	0.017 *
**Protective Factors**			
Male sex *Reference (female sex)*	−2.74	−3.89–−1.59	<0.001 ***
Employment *Reference (unemployed)*	−2.56	−4.20–−0.91	0.002 **
Student status *Reference (secondary school)*	−2.16	−3.95–−0.37	0.018 *
Income >15,000 SAR *Reference (<5000 SAR)*	−1.39	−2.67–−0.11	0.033 *
Fiber-rich foods *Reference (no consumption of specific foods/beverages)*	−1.07	−2.09–−0.06	0.037 *

* Full model included age, BMI, nationality, residence, marital status, all income categories, all education levels, physical activity, smoking status, meal frequency, and consumption of various foods and beverages, as well as chronic disease history. Model fit: R^2^ = 0.191, Adjusted R^2^ = 0.151. n = 831. Only significant predictors (p < 0.05) are shown; * p < 0.05; ** p < 0.01; *** p < 0.001. β, unstandardized regression coefficient; CI, confidence interval; GERD: Gastroesophageal reflux disease; SAR: Saudi Riyal.

**Table 8:** Predictors of GERD: Multiple logistic regression *.

Predictor	OR	95% CI	*p*-Value
NES score (per point)	1.05	1.02–1.07	0.001 **
Income 10,000–14,999 SAR * Reference (income < 5000 SAR)	2.02	1.13–3.67	0.019 *
Tea consumption *Reference (non-consumers)*	1.51	1.03–2.22	0.034 *
Asthma *Reference (non-asthma patients)*	1.79	1.01–3.10	0.042 *
Hiatal hernia *Reference (patients without hernia)*	3.61	1.24–5.93	0.030 *
Family history of GERD *Reference (patients without family history)*	1.67	1.13–2.44	0.009 **

* Full model included age, sex, nationality, residence, marital status, occupation, education, all income categories, BMI, physical activity, smoking status, meal frequency, dietary habits, and chronic disease history. Model fit: R^2^ = 0.132. n = 831. Only significant predictors (p < 0.05) are shown; * p < 0.05; ** p < 0.01;. OR: Odds ratio; CI: Confidence interval; NES: Night eating syndrome; GERD: Gastroesophageal reflux disease; SAR: Saudi Riyal.

## Data Availability

The data presented in this study is available on request from the corresponding author. The data is not publicly available due to ethical restrictions and privacy concerns related to sensitive health information.

## Abbreviations

The following abbreviations are used in this manuscript:

GERD	Gastroesophageal reflux disease
NES	Night eating syndrome
NEQ	Night eating questionnaire
GerdQ	GERD questionnaire
SAR	Saudi Riyal
